# Molecular-Dynamics Simulations of the Emergence of Surface Roughness in a Polymer under Compression

**DOI:** 10.3390/ma14237327

**Published:** 2021-11-30

**Authors:** Robin Vacher, Astrid S. de Wijn

**Affiliations:** 1 SINTEF, SINTEF Industry Materials and Nanotechnology, 7034 Trondheim, Norway; 2 Porelab and Department of Mechanical and Industrial Engineering, Norwegian University of Science and Technology, 7491 Trondheim, Norway

**Keywords:** polymer, roughness, molecular dynamics

## Abstract

Roughness of surfaces is both surprisingly ubiquitous on all length scales and extremely relevant practically. The appearance of multi-scale roughness has been linked to avalanches and plastic deformation in metals. However, other, more-complex materials have mechanisms of plasticity that are significantly different from those of metals. We investigated the emergence of roughness in a polymer under compression. We performed molecular-dynamics simulations of a slab of solid polyvinyl alcohol that was compressed bi-axially, and we characterised the evolution of the surface roughness. We found significantly different behaviour than what was previously observed in similar simulations of metals. We investigated the differences and argue that the visco-elasticity of the material plays a crucial role.

## 1. Introduction

Roughness of surfaces plays an important role in many practical settings, such as friction and adhesion (see, for example, [1]). It appears on all length scales, from geological roughness of mountain ranges, to the microscale roughness of polished surfaces, down to atomic scales. It has been shown that many surfaces display roughness in similar ways on different length scales and are close to self-affine [2,3,4,5], meaning that there is a scale invariance of the roughness, and the roughness profile looks similar. This applies to many different materials, rocks, glasses, metals, etc. Roughness of surfaces is a random, statistical property that appears naturally, with no or very minimal intentional interference.

While the self-affinity is extremely universal, we do not yet have a general understanding of why it appears. The development of roughness of a material surface involves a range of complex physical phenomena such as dislocation dynamics and crack formation (see, for example, [6,7]). In metals, for example, the surface evolution at a microscopic scale is mainly linked to the emergence of dislocations [8]. The heterogeneity of the deformation in the material across different length scales is a common mechanism cited to explain the origin of the roughness and the self-affine surface properties [7,9,10,11,12]. In order to investigate this phenomenon, Hinkle et al. [9] performed molecular-dynamics simulations of the compression of metals and showed that self-affine roughness appeared spontaneously.

Roughness appears in many other materials besides metals, including in much more complex materials. Zhang et al. showed that self-affine roughness appears in oxide glasses obtained from the cooling down of melts but that fractured surfaces are different [7]. Even more complex materials, such as polymers, are likely to have even more complex mechanisms that play a role and that involve new length scales that enter into the structure and dynamics [13]. Moreover, the self-affinity of roughness plays a crucial role in the friction of polymers [14,15].

In this work, we investigated the emergence of surface roughness in polymer surfaces. We performed molecular-dynamics simulations of solid polyvinyl alcohol (PVA). We analysed the roughness and found qualitative differences between the polymer and metals. We investigated these differences in detail and linked them to the structure and dynamics of the polymer.

## 2. Materials and Methods

### 2.1. Simulation Setup

**Figure 1:**
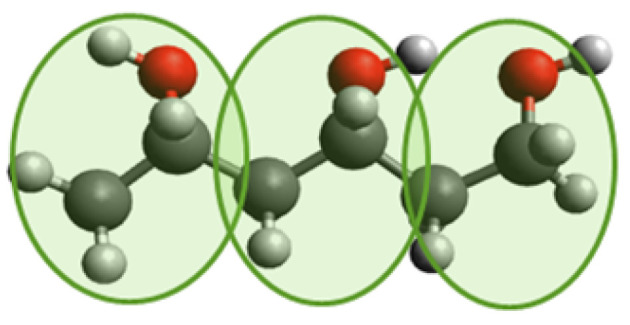
Coarse-grained model for polyvinyl alcohol (PVA) (C${}_{2}$H${}_{4}$O)${}_{x}$. Red atoms are oxygen; dark gray are carbon; and light gray are hydrogen. One green circle represents one coarse-grained particle that replaces the group of atoms C${}_{2}$H${}_{4}$O. Each monomer contains two carbons from the backbone. The model has harmonic stretching and bending provided in tabular form.

We used a non-crosslinked polymer, polyvinyl alcohol (PVA), to create a solid polymer substrate. The approach is similar to that in our previous work [16]. The PVA was described using a coarse-grained force field developed by Müller–Plathe et al. [17]. Each polymer particle represents one structural unit of C${}_{2}$H${}_{4}$O (see Figure 1). The interaction between monomers consists of bonded and non-bonded contributions. The non-bonded interaction is given by a Lennard–Jones 96 potential $V_{\mathrm{pair}}\left(r\right)=4\epsilon_{0}\left[{\left(\frac{\sigma_{0}}{r}\right)}^{9}-{\left(\frac{\sigma_{0}}{r}\right)}^{6}\right]$ where $\epsilon_{0}=0.0179$ eV is the depth of the potential, $\sigma_{0}=4.628\;Å$ the distance at which the potential vanishes, and *r* is the distance between the monomers. The stretching of a bond is described by a harmonic potential $V_{\mathrm{bond}}=K{\left(r-r_{0}\right)}^{2}$ where $K=2.37\;eV/{Å}^{2}$ characterizes the stiffness of a spring, and $r_{0}=2.6\;Å$ is the equilibrium bond length. The bending potential was approximated by an angular potential, which is provided in table format. The system was integrated using the Velocity Verlet algorithm, and the time step was set to 0.8 fs. The simulations were performed using LAMMPS [18].

A solid polymer sample was created by quenching a polymer melt. The melt was set up using the same approach as described in [16]. We simulated 10 million particles in chains of 50. Our box had a length in *x* and *y* of 83 nm and was much larger in the *z* direction. Before solidification, two repulsive walls at a distance of 200 nm apart and perpendicular to the *z* direction were used to contain the melt.

**Figure 2:**
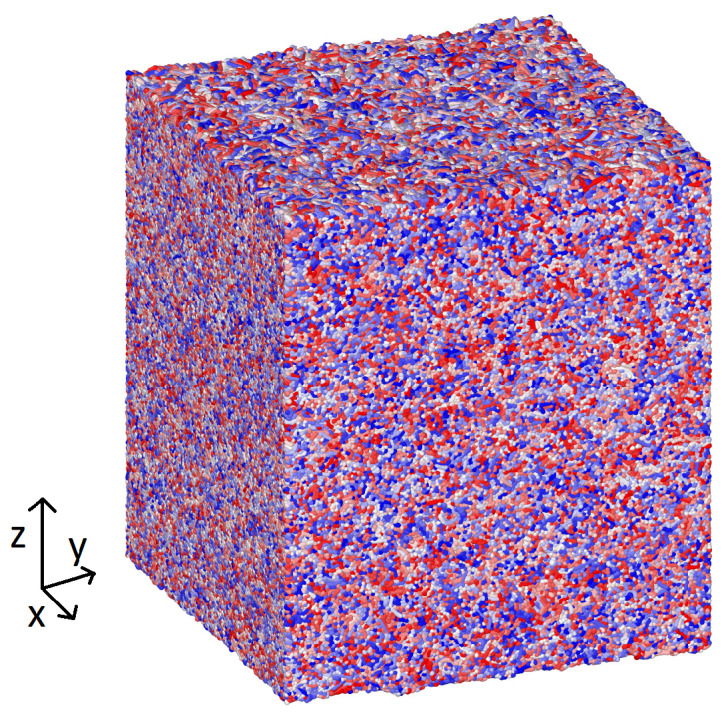
Polymer substrate after the cooling process and before compression. The colours represent different chains of the polymer. The simulation box had periodic boundary conditions in the *x* and *y* direction, with, initially, a length of 83 nm. It was extended in the *z* direction. Initially, the height of the slab was about 95 nm. Once we obtained this sample, we compressed it in the *x* and *y* directions, and it expanded in *z*, while becoming rougher.

The temperature of the melt was controlled using a Nosé–Hoover thermostat with a damping parameter equal to 0.16 ps. Initially, the melt was equilibrated at a temperature of 2000 K for 5 ns. Then, the temperature was reduced gradually to 270 K with a cooling rate equal to 216 K/ns. This produced a slab of solid semi-crystalline material, as is shown in the snapshot in Figure 2. The walls in the *z* direction could then be safely removed.

Once we had created the solid slab of polymer, for our investigations of the emerging roughness, we compressed the material equi-biaxially with a strain $\epsilon =\Delta L/L_{0}$ of up to 40% and a strain rate of 0.09375/ns. This was achieved by rescaling the system in the *x* and *y* but not *z* directions. During this compression the temperature was kept constant at 270 K using the same Nosé–Hoover thermostat.

### 2.2. Calculation of the Self-Affinity of the Roughness under Magnification

As we compressed our simulated polymer block, we analysed the evolution of the roughness of the surface. We followed the approach of Hinkle et al. [9] and calculated a Hurst exponent [19] for the roughness profile, to characterise its self-affinity.

We defined the height of a section of the surface down to the resolution of 1 LJ unit $\sigma_{0}$. We divided the box in the *x* and *y* directions into bins of this size. The height in each bin was taken as the position of the highest monomer in each bin. The surface roughness $S_{q}$ was then calculated as the variance of the height, i.e., $S_{q}=\sqrt{}\left[\frac{1}{N_{\mathrm{bins}}}\sum_{i=1}^{N_{\mathrm{bins}}}{\left(Z_{i}-\bar{Z}\right)}^{2}\right]$, where the sum runs over all $N_{\mathrm{bins}}$ bins involved in the section of the surface under consideration; $Z_{i}$ is the highest *z* coordinate of all particles in bin *i*; and $\bar{Z}$ is the average over all bins in the section.

We investigated the self-affinity by considering the surface under different magnification ζ and splitting the surface into ζ×ζ smaller sections. The roughness was calculated for every section, and this value was averaged over all sections.

We then considered the dependence of the average roughness on the magnification. If the surface is self-affine, this is a power-law. The exponent of this power-law, the Hurst exponent, gives the scaling of the self-affinity. We estimated the Hurst exponent of this dependence via a least square linear fit taken between a magnification of 2 and 60. Higher magnifications above 60 are not meaningful, as the size of each section becomes comparable to $\sigma_{0}$.

### 2.3. Calculation of the Nodal Displacements 

To investigate the visco-elasticity during compression, we characterised the displacement of monomers relative to their neighbours and used it to determine the plasticity. We measured the changes in the distance between neighbouring particles. Particles that are at a distance less than $2\sigma_{0}$ were considered neighbours. After a change in strain of 0.0375, the distances between a particle and its neighbours changed. A probability distribution function (PDF) was computed to elucidate how the changes in the distance evolve during the compression. If the distance between two neighbouring particles increases by more than a specific threshold amount, then we assumed they will no longer return directly to each other’s vicinity when the strain is reversed. We used a value of $1.5\sigma_{0}$ as the threshold that defines when a pair is considered to have a reversible elastic or an irreversible deformation. When the variation of distance of a pair of particles is above that limit, then this pair is counted as having an irreversible (plastic) deformation. The ratio of the plastic pairs over the total number of pairs provides the level of plasticity. We restricted ourselves here to direct elastic deformation with monomers returning to their original positions when the strain was reduced. In principle, also entropic contributions to the elasticity are possible. However, in glasses, which are not in equilibrium, this is poorly defined.

We note that we did not distinguish further based on how the irreversible deformation depends on the deformation rate, i.e., if it is viscous or not. In glassy materials like polymers, there is usually some kind of rate dependence that can be quite complex.

## 3. Results

**Figure 3:**
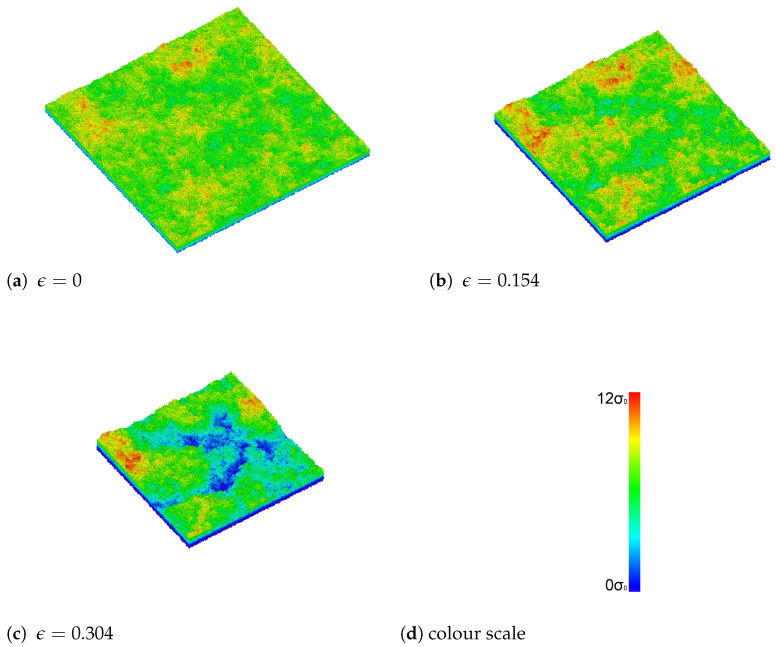
Snapshots of the surface during the biaxial compression, for (**a**) no strain, (**b**) ϵ=0.154, and (**c**) ϵ=0.304. (**d**) is the colour scale. The colour represents the height of the surface particles compared to the highest one. The same colour scale was used of $12\sigma_{0}$ between red and blue. As the sample is compressed, the surface area becomes smaller and the roughness increases.

**Figure 4:**
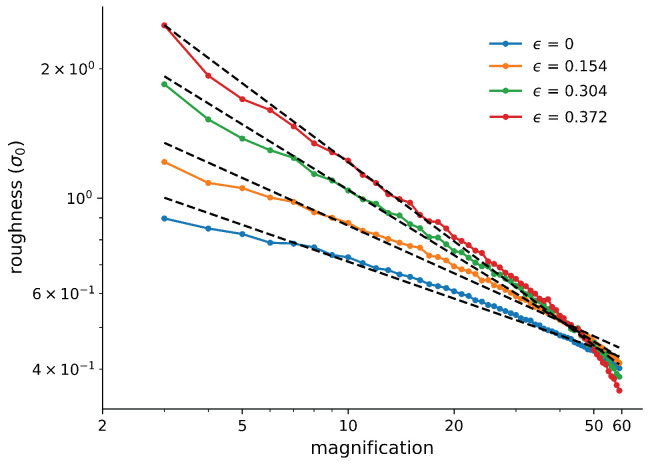
Log-log plot of the roughness versus magnification for different strains. The Hurst exponent is the exponent of the power-law dependence. High compression leads to higher roughness as well as an increase in the Hurst exponent. Details on how the roughness was calculated and how the magnification was defined can be found in Section 2.2.

During compression, our sample becomes visibly rougher, as expected. This can be seen in Figure 3, which shows snapshots of the surface at different values of the strain. Without strain, there was a small initial roughness. To quantify the self-affinity of the evolving roughness profile, we calculated the surface roughness at different magnifications. This is shown in Figure 4 for several different strain values. The slope of the curves gives the Hurst exponent of the roughness. The surface had an initial non-vanishing roughness and self-affinity. While initially the melt was confined by flat walls, it shrunk as it cooled, and the walls were far away from the final surfaces. The cooling down of the melt already produced self-affine roughness on the surface. Similar behaviour has also been observed in atomic-scale simulations of other glassy materials [7].

**Figure 5:**
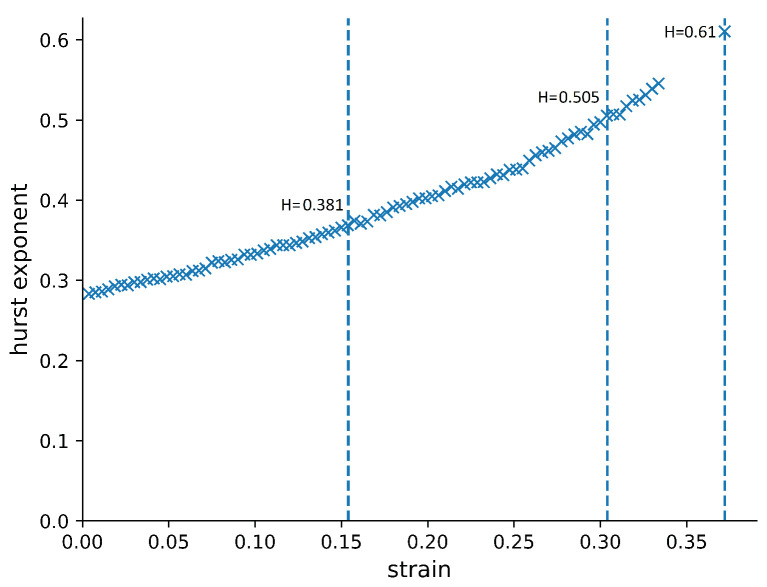
The Hurst exponent of the surface roughness of the compressed polymer sample as a function of the strain. The Hurst exponent continued to increase with increasing strain, rather than levelling off to a constant value, as it has been shown to do in metallic materials [9]. The dashed lines are here to underline the values of the Hurst exponent at different strains.

In Figure 5, we show how the Hurst exponent develops with the strain. It clearly increases gradually but does not reach a plateau. This is qualitatively different from what has been found for metallic materials [9], where the Hurst exponent levels off around strain 0.1 and converges to a value around 0.4. This qualitatively different behaviour must be related to the qualitatively different dynamical and structural properties of the polymer. This could lead to time-dependent structure changes, combined with the critical slowdown of equilibration due to glassiness, which would not appear in metallic systems. In order to investigate this further, we analysed the structure of the polymer in our simulations. This will allow us to draw conclusions about polymers in general, beyond what happens in just PVA.

**Figure 6:**
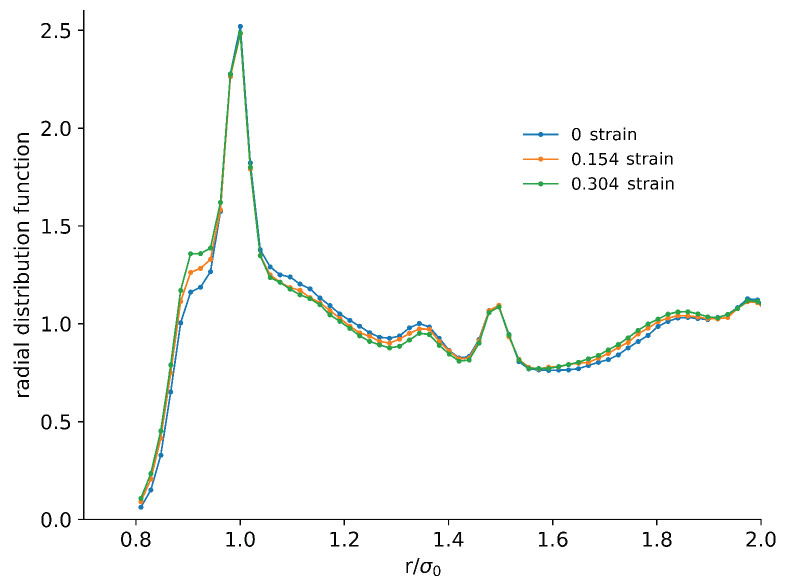
Radial distribution function of the monomers for three different values of the strain, as a measure of the internal structure in the material. There were quantitative differences in the height of the peaks for the different strains, but there were no qualitative structural changes.

We first characterised the structure using the radial distribution function (RDF), which is shown in Figure 6. The large peak around 1.0 corresponds to the bonds inside the polymer chain and was not changed significantly during compression, as we did not allow for bond breaking. At shorter distances, around 0.9, there was a shoulder that resulted from non-bonded monomers approaching each other quite closely. This increased in height with increasing strain. Correspondingly, the density decreased at the slightly longer distances in the range of $1.1\sigma_{0}$–$1.4\sigma_{0}$. Further out, there was an increase. However, the peaks remained in place and were qualitatively similar. These changes in the RDF are indicative of distortion in the material but not any dramatic structural changes.

**Figure 7:**
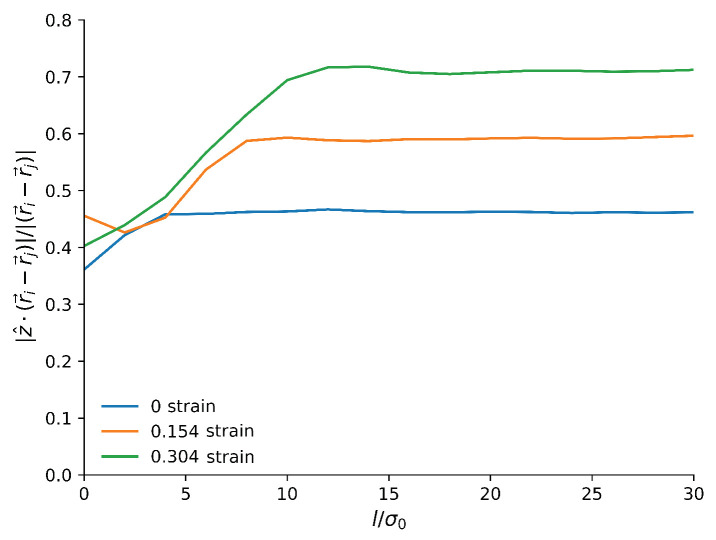
Average normalized component of the bond in the *z* direction as a function of the *z* position in the slab relative to the highest particle, for three different strains. As the sample is deformed the bonds become more anisotropic, aligning with the direction that is stretching.

As our compression was anisotropic, it is possible that anisotropic structural changes may appear that would not be picked up in the RDF. We therefore investigated anisotropy by considering the mean component of the monomer–monomer bonds in the *z* direction. This is shown in Figure 7.

We can see that close to the surface, from the beginning, the chains tend to be aligned parallel to the surface, as expected. Before compression, in the bulk, the average *z* component was around 0.47, which is close to the value expected from purely random directions, $\frac{1}{2}$. The small difference was likely due to the finite size of our sample and the long-range effects of the boundaries. The average *z* component of the bonds increased during the compression, indicating that there is a vertical reorientation of the chains. These results clearly indicate that the polymer, once compressed, is no longer isotropic.

Now that we have established that there is distortion of the structure in the polymer, we consider the dynamics in more detail. Deformation of materials can generally be described as a combination of elastic (reversible) and plastic (irreversible). In polymers, elastic deformation can be quite large, unlike in metals, where, for large deformations, plasticity dominates. We investigated this in our system through the nodal displacement (see Section 2.3), which describes how many monomers have left their original environment of neighbouring particles.

**Figure 8:**
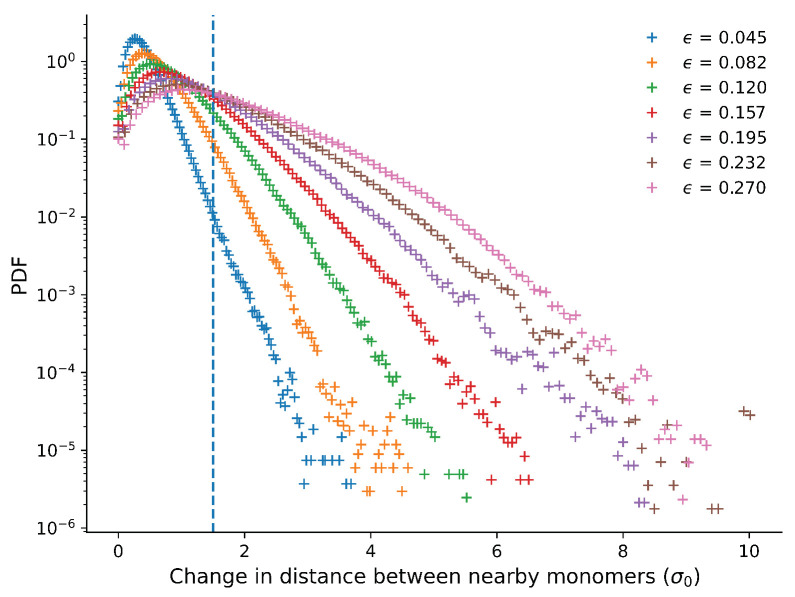
The probability distribution function of the relative displacements of monomers for several different values of the strain. The vertical line represents the limit displacement at which a pair of monomers are considered to have been plastically displaced.

Figure 8 shows the distribution of nodal displacements for different strains. We can see that the nodal displacements increased during the compression and especially that bigger displacements become much more likely for larger strains. An important point to notice in this plot is that the tails of the distributions are exponential for all strains shown. This means that on the length scale of our simulations, there is no power-law distribution of rearrangements. This further indicates that different mechanisms are at play in our system on different scales, rather than a single mechanism that acts on all length scales, which would produce scale-free behaviour in the form of a power-law. We suspect that on small scales, rearrangements of single monomers are dominant, while the length of the polymers and size of the crystal grains become important on larger scales.

One obvious distinction between displacement mechanisms that occur in this system, but not to the same degree in metallic systems, is the combination of significant elastic and plastic deformation. We can quantify the contributions from the different mechanisms through the magnitude of the nodal displacements. Elastic deformation would distort the neighbourhood but not remove monomers from their neighbourhoods. High nodal displacements are therefore related to plasticity, while low nodal displacements indicate that the distortion is elastic. We considered a node plastically displaced if the nodal displacement exceeds $1.5\sigma_{0}$ over a change in total strain of 0.0375, i.e., enough to have left the energy minima in the non-bonded potential of their neighbours.

**Figure 9:**
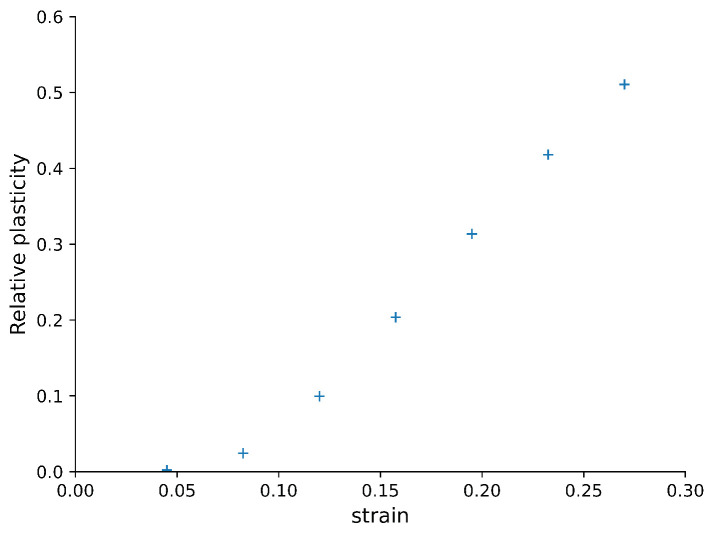
Relative plasticity, i.e., the fraction of monomer pairs that displayed plastic local changes. We defined plasticity in terms of the change in distance between monomers. The change in distance was recorded between two states with a 0.0375 difference in strain. A pair of monomers was considered to have moved plastically if the change in the distance between them is more than $1.5\sigma_{0}$.

Figure 9 shows the fraction of neighbour pairs that have been displaced plastically, as a function of the strain. At the beginning of the compression, there was almost no irreversible plastic deformation, i.e., all deformation was elastic. Then, there was a transition where plastic deformation started to appear around 0.08 strain. At large strains, more than 70% of the monomers are part of plastic deformation. Plastic flow has taken over.

**Figure 10:**
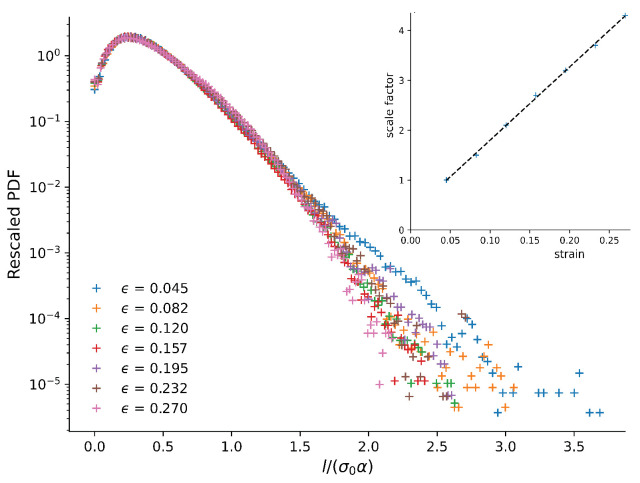
Rescaled PDF vs rescaled nodal distance presented in a semi-log plot. The scaling parameter was linear in the strain and obtained from a linear fit (see inset) of the ratio of the nodal displacements corresponding to the maximum in the PDF, 14.667×ϵ+0.33. For large strains, the tail of the distribution was exponential, indicating that the mechanism involved acts on a limited length scale.

To investigate if the nature of the plasticity changed significantly during the compression, we considered the shape of the distribution of nodal displacements. We rescaled the nodal displacement and distribution shown in Figure 8 by a scale factor α that is linear in the strain. We obtained this linear function by considering the nodal displacements corresponding to the maximum in the PDF and fitting a linear function, which gives 14.667×ϵ+0.33. The result is shown in Figure 10. For the most common displacements, the curves fall neatly on top of each other. However, the curve for the small strain is different from the others, as expected from the fact that at small strains, elastic deformation dominates, while at larger strains, both elastic and plastic deformation occur.

**Figure 11:**
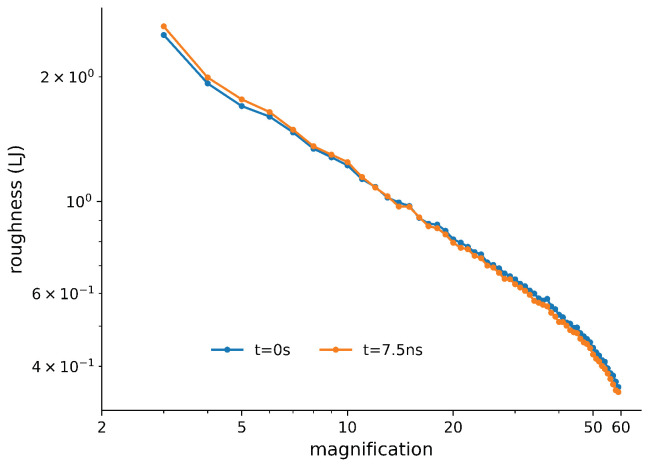
Roughness versus magnification when the compression was stopped at a strain of ϵ=0.372. The blue curve corresponds to the moment when the compression was stopped, while the orange curve was after 7.5 ns of relaxation. There are changes in the roughness at both high and low magnification.

Finally, we considered the time scale of the dynamics of rearrangements and changes inside the material and how they affect the roughness. While we cannot probe significantly different strain rates due to limitations in available computing power, we can explore the time scales by allowing a strained substrate to relax without further compression and observing changes in the roughness over time. We stopped the compression at a strain of 0.372. We then ran the simulation with a constant box size for 7.5 ns. A comparison of the roughness before and after is shown in Figure 11. We could observe a decrease in the surface roughness at high magnification and an increase at low magnification. This suggests that there are dynamic processes still going on at the surface or inside the bulk of the material on all length scales. Since there was nothing special about the strain of 0.372, we expected that similar behaviour would appear if we stopped the compression earlier or later.

## 4. Discussion

It is clear from our results that the polymer in our simulations does not produce self-affine roughness in the same way that metallic materials do. Unlike in the case of metals, under compression, the Hurst exponents of our polymer surfaces did not converge to a constant value. This means that the nature of the roughness continued to change.

In order to understand the reasons for this behaviour and to be able to draw general conclusions, we investigated this difference in more detail through a number of indicators of structure. As the material is compressed, we observed changes in structural properties, such as the radial distribution function and the average alignment of bonds. These structural properties continued to change and did not reach a plateau. This indicates that the material itself continues to change, and therefore it is not surprising that mechanical properties such as the distribution of rearrangements also continue to change during further compression, which in turn can affect the formation of the surface roughness.

A crucial difference between metals and polymers that plays a role here is the visco-elasticity. The elastic deformation of the polymer in our simulations was significant compared to the total strain, which means that even at high strain, when plastic strain dominates completely in metals, there is residual elastic strain in our polymer. This combination of elastic and plastic strain shifts during the compression, giving a strain-dependence to the mechanical properties.

We also considered the rearrangements in the structure that occurred during the compression. In general, a power-law distribution of rearrangements would be expected to be linked to avalanches in rearrangements [20,21,22], as well as self-affine roughness profiles [9]. We therefore investigated the displacement of monomers from their environment. While we would not expect long-range power-law behaviour here, due to the finite size of our simulation box, we did not observe any power-law at all (see Figure 10). This may be related to the fact that the displacements are in fact within the length of the polymer, and any power-law rearrangements would have to include the entire chain.

Finally, we consider the dynamics. Our polymer was glassy, which means that dynamics may occur on very long time scales. In the simulation where we stopped compressing, the material continued to change, reducing the roughness on small length scales while also increasing it on longer length scales. It may be that small-scale surface flow is occurring, which smooths out the surface. Meanwhile, large, long-time glassy rearrangements in the bulk material could be producing higher roughness on larger scales. Hinkle et al. [9] observed a temperature dependence of the emerging Hurst exponent in their simulations of metallic glasses, which also suggests dynamic (thermal) relaxation effects.

It would be extremely desirable to compare our results to experiments on materials surfaces during compression. However, to our knowledge, such experiments are not yet being performed anywhere. We hope that our simulation results, and those of Hinkle et al., will stimulate experimental investigations of the emergence of roughness on atomic scales.

From all of the above, it is clear that some of the remaining questions about this system could be resolved if we could significantly increase the size of our simulation box. We are however limited in the length scales that we can achieve, due to limitations in computational power. The simulations we presented in this work contained 10 million coarse-grained particles, and the full compression takes around 0.2 million CPUcore hours to run. An order of magnitude larger range of length scales would require a larger simulation box, with three orders of magnitude more particles, which would become prohibitively computationally expensive. Similarly, repeating these simulations for a number of other polymers would be computationally very expensive as well, especially since many polymers have electrostatic interactions, which by their long-range nature slow down simulations considerably.

## 5. Conclusions

We investigated the emergence of roughness and its scale invariance in polymers using molecular-dynamics simulations, by compressing a large slab of material. We found qualitative differences when compared to metals. The Hurst exponent, which quantifies the self-affinity, continues to change with increasing strain. We attributed this to the visco-elastic properties of the polymer combined with structural changes in the material, such as anisotropy resulting from anisotropic stresses. We further investigated the structural changes and dynamics during compression. We found, in addition, that there are long time scales involved in the dynamics, and the roughness continued to evolve when the compression was stopped.

## Data Availability

Not applicable.
